# Supplementary material for: Association between self-reported napping and risk of cardiovascular disease and all-cause mortality: A meta-analysis of cohort studies
Source: PLoS One. 2024 Oct 16;19(10):e0311266. doi: 10.1371/journal.pone.0311266 (PMC11482734; doi:10.1371/journal.pone.0311266)
Supplement: S1 Table — (DOCX) [file pone.0311266.s001.docx]

| **Section and Section and Topic** | **Item** | **Checklist item** | **Location where item is reported** |
| --- | --- | --- | --- |
| **TITLE** | | | |
|  | 1 | Association between self-reported napping and risk of cardiovascular disease and all-cause mortality: A meta-analysis of cohort studies | Manuscript page 1 |
| **ABSTRACT** | | | |
| Abstract | 2 | Objectives: This meta-analysis aims to assess the association between adult nap duration and risk of all-cause mortality and cardiovascular diseases（CVD）.  Methods: PubMed, Cochrane Library, Embase and Web of Science databases were searched to identify eligible studies. The quality of observational studies was assessed using the Newcastle-Ottawa Scale. We performed all statistical analyses using Stata software version 14.0. For the meta-analysis, we calculated hazard ratio (HR) and their corresponding 95% confidence intervals (CIs). To assess publication bias, we used a funnel plot and Egger's test.  Results: A total of 21 studies involving 371,306 participants revealed varying methodological quality, from moderate to high. Those who indulged in daytime naps faced a significantly higher mortality risk than non-nappers (HR: 1.28; 95% CI: 1.18-1.38; I2=38.8%; P＜0.001). Napping for less than 1 hour showed no significant association with mortality (HR: 1.00; 95% CI: 0.90-1.11; I2=62.6%; P=0.971). However, napping for 1 hour or more correlated with a 1.22-fold increased risk of mortality (HR: 1.22; 95% CI: 1.12-1.33; I2=40.0%; P＜0.001). The risk of CVD associated with napping was 1.18 times higher than that of non-nappers (HR: 1.18; 95% CI: 1.02-1.38; I2=87.9%; P=0.031). Napping for less than 1 hour did not significantly impact CVD risk (HR: 1.03; 95% CI: 0.87-1.12; I2=86.4%; P＝0.721). However, napping for 1 hour or more was linked to a 1.37-fold increased risk of CVD (HR: 1.37; 95% CI: 1.09-1.71; I2=68.3%; P＝0.007).  Conclusions: Our meta-analysis indicates that taking a nap increases the risk of overall mortality and CVD mortality. It highlights that the long duration time of the nap can serve as a risk factor for evaluating both overall mortality and cardiovascular mortality. | Manuscript page 1-2 |
| **INTRODUCTION** | | | |
| Rationale | 3 | Napping, also known as a daytime nap or short rest, involves taking a brief period of relaxation during the day. Nap can mitigate health risks associated with insufficient sleep. Moreover, napping has been shown to enhance attention, improve work efficiency, and reduce stress. Research indicates that while napping can be a risk factor for cancer and obesity, it also serves as a protective factor against cardiovascular disease (CVD), all-cause mortality, depression, and diabetes. However, the duration of napping appears pivotal in determining its effects. Exploring the relationship between napping, CVD, and all-cause mortality is crucial for predicting risk factors effectively and improving overall health outcomes. However, previous systematic reviews and meta-analyses have predominantly focused on nighttime sleep duration in relation to overall mortality, CVD, and associated risks. The connection between daytime napping and overall mortality, as well as cardiovascular risk factors, remains inconclusive. A meta-analysis conducted in 2015, comprising seven studies, suggested that napping could predict overall mortality but not cardiovascular mortality. Contrarily, recent meta-analytical findings from 2020 diverge from earlier studies and omit seven recent publications. Given the conflicting conclusions from these newly published studies and existing meta-analyses, the current evidence base may not be the most up-to-date. Therefore, this study aims to provide a comprehensive review of the latest evidence concerning the association between adult nap duration and both overall mortality and CVD. | Manuscript page 3 |
| Objectives | 4 | The objective of this review is provide a comprehensive review of the latest evidence concerning the association between adult nap duration and both overall mortality and CVD | Manuscript page 3 |
| **METHODS** | | | |
| Eligibility criteria | 5 | Exclusion criteria included: reports, reviews, conference abstracts, and studies reporting duplicate results; lack of odds ratio (OR) and hazard ratio (HR) estimates with corresponding 95% confidence intervals (CI). | Manuscript page 4 |
| Information  sources | 6 | The review included studies published between 1^st^ January 2013 and 1^st^ March 2023. This systematic review was conducted using five computerized databases, which include PubMed, Scopus, Embase, Cochrane Library and ProQuest. | Manuscript page 4 |
| Search strategy | 7 | **PubMed:**  ((((((((((Napping[Title/Abstract]) OR (Nap[Title/Abstract])) OR (Daytime sleep[Title/Abstract])) OR (Siesta[Title/Abstract])) OR (Daytime sleepiness[Title/Abstract])) OR (Daytime somnolence[Title/Abstract])) OR (Dozing[Title/Abstract])) OR (Catnap[Title/Abstract])) OR (Snooze[Title/Abstract])) AND (("Mortality"[Mesh]) OR ((((((((((Mortality[Title/Abstract]) OR (Death[Title/Abstract])) OR (suicide[Title/Abstract])) OR (Case Fatality[Title/Abstract])) OR (Crude Death[Title/Abstract])) OR (Mortality Decline[Title/Abstract])) OR (Mortality Determinant[Title/Abstract])) OR (Differential Mortality[Title/Abstract])) OR (Age-Specific Death Rate[Title/Abstract])) OR (Excess Mortality[Title/Abstract])))) AND (("Cardiovascular Diseases"[Mesh]) OR ((((((((((((Cardiovascular Diseases[Title/Abstract]) OR (Cardiac Event[Title/Abstract])) OR (Cardiomyopathy[Title/Abstract])) OR (heart failure[Title/Abstract])) OR (cerebrovascular disease[Title/Abstract])) OR (peripheral vascular disease[Title/Abstract])) OR (coronary heart disease[Title/Abstract])) OR (ischemic heart disease[Title/Abstract])) OR (myocardial infarction[Title/Abstract])) OR (stroke[Title/Abstract])) OR (heart attack[Title/Abstract])) OR (hypertension[Title/Abstract]))).  **Embase:**  #1: 'napping'/exp OR napping OR nap:ab,ti OR 'daytime sleep':ab,ti OR siesta:ab,ti OR 'daytime sleepiness':ab,ti OR 'daytime somnolence':ab,ti OR dozing:ab,ti OR catnap:ab,ti OR snooze:ab,ti  #2: 'mortality'/exp  #3: 'mortality'/exp OR mortality OR death:ab,ti OR 'daytime sleep':ab,ti OR 'case fatality':ab,ti OR 'crude death':ab,ti OR 'mortality decline':ab,ti OR 'mortality determinant':ab,ti OR 'differential mortality':ab,ti OR 'age-specific death rate':ab,ti OR 'excess mortality':ab,ti  #4: #2 OR #3  #5: 'cardiovascular disease'/exp  #6: 'cardiovascular diseases'/exp OR 'cardiovascular diseases' OR (('cardiovascular'/exp OR cardiovascular) AND ('diseases'/exp OR diseases)) OR 'cardiac event':ab,ti OR cardiomyopathy:ab,ti OR 'heart failure':ab,ti OR 'cerebrovascular disease':ab,ti OR 'peripheral vascular disease':ab,ti OR 'coronary heart disease':ab,ti OR 'ischemic heart disease':ab,ti OR 'myocardial infarction':ab,ti OR stroke:ab,ti OR 'heart attack':ab,ti OR hypertension:ab,ti  #7: #5 OR #6  #8: #2 AND #4 AND #7  **Cochrane Library**  #1: (Napping):ti,ab,kw OR (Nap):ti,ab,kw OR (Daytime sleep):ti,ab,kw OR (Siesta):ti,ab,kw OR (Daytime sleepiness):ti,ab,kw  #2: (Daytime somnolence):ti,ab,kw OR (Dozing):ti,ab,kw OR (Catnap):ti,ab,kw OR (Snooze):ti,ab,kw  #3: #1 OR #2  #4: MeSH descriptor: [Mortality] explode all trees  #5: (Mortality):ti,ab,kw OR (Death):ti,ab,kw OR (suicide):ti,ab,kw OR (Case Fatality):ti,ab,kw OR (Crude Death):ti,ab,kw  #6: (Mortality Decline):ti,ab,kw OR (Mortality Determinant):ti,ab,kw OR (Differential Mortality):ti,ab,kw OR (Age-Specific Death Rate):ti,ab,kw OR (Excess Mortality):ti,ab,kw  #7: #4 OR #5 OR #6  #8: MeSH descriptor: [Cardiovascular Diseases] explode all trees  #9: (Cardiovascular Diseases):ti,ab,kw OR (Cardiac Event):ti,ab,kw OR (Cardiomyopathy):ti,ab,kw OR (heart failure):ti,ab,kw OR (cerebrovascular disease):ti,ab,kw  #10: (peripheral vascular disease):ti,ab,kw OR (coronary heart disease):ti,ab,kw OR (ischemic heart disease):ti,ab,kw OR (myocardial infarction):ti,ab,kw OR (stroke):ti,ab,kw  #11: (heart attack):ti,ab,kw OR (hypertension):ti,ab,kw  #12: #8 OR #9 OR #10 OR #11  #13: #3 AND #7 AND #12  **Web of science:**  #1: Napping (Topic) or Nap (Topic) or Daytime sleep (Topic) or Daytime sleepiness (Topic) or Siesta (Topic) or Daytime somnolence (Topic) or Dozing (Topic) or carnap (Topic) or sneeze (Topic)  #2: Mortality (Topic) or Death (Topic) or suicide (Topic) or Case Fatality (Topic) or Crude Death (Topic) or Mortality Decline (Topic) or Mortality Determinant (Topic) or Differential Mortality (Topic) or Age-Specific Death Rate (Topic) or Excess Mortality (Topic)  #3: Cardiovascular Diseases (Topic) or Cardiac Event (Topic) or Cardiomyopathy (Topic) or heart failure (Topic) or cerebrovascular disease (Topic) or peripheral vascular disease (Topic) or coronary heart disease (Topic) or ischemic heart disease (Topic) or myocardial infarction (Topic) or stroke (Topic) or heart attack (Topic) or hypertension (Topic)  #4: #1 AND #2 AND #3 | Retrieval strategies document |
| Selection process | 8 | Two researchers independently screened the literature based on the inclusion and exclusion criteria. After removing duplicates, the initial screening phase involved evaluating titles and abstracts. In the second phase, full texts of potentially eligible articles were reviewed to confirm adherence to the criteria. Any disagreements among the researchers were resolved through discussion with a third researcher. | Manuscript page 4 |
| Data collection  process | 9 | Two researchers followed established guidelines for data extraction and used a pre-designed table for this purpose. The extracted data included details such as the first author. Any discrepancies were resolved by consulting a third researcher. | Manuscript page 4 |
| Data items | 10 | The data were extracted using a pre-designed form encompassing the following: publication year, country, follow-up duration, sample size, cohort characteristics, age distribution, assessment of daytime napping, subgroup analysis, adjustment for confounding factors, and NOS scores | Manuscript page 4-5 |
| Study risk of bias  assessment | 11 | Based on the Newcastle-Ottawa Scale (NOS), two researchers assessed the quality of cohort studies, considering three key factors: participant selection for exposed and unexposed groups, comparability between the groups, and outcome assessment. The total NOS score ranges from 0 to 9 points. Out of the articles included in this study, 6 were classified as having moderate quality, while the remaining articles were deemed high quality. | Manuscript page 5 |
| Effect measures | 12 | prevalence of CVD and the overall risk ratio (HR) of all-cause mortality between the napping and non-napping populations. | Manuscript page 5 |
| Synthesis  methods | 13 | We conducted a meta-analysis using the DerSimoniane-Laird random-effects model, comparing the prevalence of CVD and the overall risk ratio (HR) of all-cause mortality between the napping and non-napping populations. The non-napping group was chosen as the reference category. In cases where multiple adjusted estimates were reported in a study, we selected the estimate with the most adjustments. Additionally, we performed subgroup analysis based on gender. Sensitivity analysis was conducted to assess the robustness of the overall results. Publication bias was evaluated using a funnel plot and Egger's test(25). All statistical analyses were performed using Stata software version 14.0. | Manuscript page 5 |
| Reporting bias  assessment | 14 | Not applicable | Not applicable |
| Certainty  assessment | 15 | Not applicable | Not applicable |
| **RESULTS** | | | |
| Study selection | 16 | Fig. 1 Flow diagram of study selection and exclusion | Manuscript page 9 |
| Study  characteristics | 17 | Table 1 Characteristics of the cohort studies included in the meta-analysis | Manuscript 18-23 |
| Risk of bias in  studies | 18 | Table 1 Characteristics of the cohort studies included in the meta-analysis | Manuscript 18-23 |
| Results of  individual studies | 19 | Table 1 Characteristics of the cohort studies included in the meta-analysis | Manuscript page 18-23 |
| Results of  syntheses | 20a | Table 1 Characteristics of the cohort studies included in the meta-analysis | Manuscript page 18-23 |
|  | 20b | Figure 2 illustrates the forest plots for the association between daytime napping and all-cause mortality, as well as CVD risk.  Figure 3 Funnel plot showing the effect of different nap durations on all-cause mortality and CVD |  |
|  | 20c | Not applicable | Not applicable |
|  | 20d | Supporting Information | Sensitivity analysis document |
| Reporting biases | 21 | Not applicable | Not applicable |
| Certainty of  evidence | 22 | Not applicable | Not applicable |
| **DISCUSSION** | | | |
| Discussion | 23a | This meta-analysis comprises 21 cohort studies involving 371,306 individuals, offering a comprehensive assessment of the relationship between napping and both overall mortality and CVD. We identified statistically significant associations indicating increased risks of overall mortality and CVD among adults who nap compared to those who do not. Specifically, napping was associated with a 1.28-fold higher risk of overall mortality and a 1.18-fold higher risk of CVD. Furthermore, napping for less than 1 hour did not show a significant association with either outcome. In contrast, napping for 1 hour or more was linked to a 1.22-fold higher risk of overall mortality and a 1.37-fold higher risk of CVD compared to non-nappers. Interestingly, gender did not influence the observed associations between daytime napping and the risks of overall mortality or cardiovascular disease. | Manuscript page 11-14 |
|  | 23b | Our meta-analysis consolidates current evidence on the association between napping and overall mortality rate as well as CVD risk, underscoring the significance of adult nap duration in early detection of these health outcomes. However, this study is subject to certain limitations. We exclusively analysed cohort studies; future investigations could benefit from integrating case-control and cross-sectional studies to diversify study methodologies. Moreover, variations in lifestyle factors and sleep habits across different countries might influence research findings. Additionally, our meta-analysis did not incorporate covariate analysis, although the cohort studies included in our review controlled for confounding variables, ensuring the robustness of our conclusions. It is important to acknowledge that reliance on interviews or questionnaires to assess nap duration could introduce measurement errors and recall biases.  This meta-analysis indicates that prolonged napping is associated with increased risks of overall mortality and cardiovascular disease. However, further research is necessary to confirm the underlying pathophysiological mechanisms involved, which could include longitudinal observational studies and genetic investigations. Additionally, to assess whether nap duration can reliably predict the risks of overall mortality and cardiovascular disease, more prospective studies involving adults are needed. | Manuscript page 14-15 |
|  | 23c |  | Manuscript page 14-15 |
|  | 23d |  | Manuscript page 14-15 |
| **OTHER INFORMATION** | | | |
| Registration and  protocol | 24a | The review already registered, Title: Association between self-reported napping and risk of cardiovascular disease and death: A meta-analysis of cohort studies. ID: CRD42024547547 |  |
|  | 24b | protocol was not prepared |  |
|  | 24c | Title changed from “Association between self-reported napping and risk of cardiovascular disease and death: A meta-analysis of cohort studies” to “Association between self-reported napping and risk of cardiovascular disease and all-cause mortality: A meta-analysis of cohort studies” |  |
| Support | 25 | Non-financial support for the review |  |
| Competing  interests | 26 | No conflict of interest has been declared by the author(s) |  |
| Availability of  data, code and  other materials | 27 | Data availability：The authors confirm that the data supporting the findings of this study are available within the article.  Code availability：Not applicable  The data related to the study are publicly available and are provided in the form of an annex. |  |
