# Supplementary material for: Association between self-reported napping and risk of cardiovascular disease and all-cause mortality: A meta-analysis of cohort studies
Source: PLoS One. 2024 Oct 16;19(10):e0311266. doi: 10.1371/journal.pone.0311266 (PMC11482734; doi:10.1371/journal.pone.0311266)
Supplement: S2 Table — (DOCX) [file pone.0311266.s002.docx]

**The retrieval strategies and retrieval results of each database are shown in Tables 1-4**.

Table 1: PubMed

| No. | Content | Result |
| --- | --- | --- |
| #1 | Search: ((((((((Napping[Title/Abstract]) OR (Nap[Title/Abstract])) OR (Daytime sleep[Title/Abstract])) OR (Siesta[Title/Abstract])) OR (Daytime sleepiness[Title/Abstract])) OR (Daytime somnolence[Title/Abstract])) OR (Dozing[Title/Abstract])) OR (Catnap[Title/Abstract])) OR (Snooze[Title/Abstract]) | [18,392](https://pubmed.ncbi.nlm.nih.gov/?term=%28%28%28%28%28%28%28%28Napping%5BTitle%2FAbstract%5D%29+OR+%28Nap%5BTitle%2FAbstract%5D%29%29+OR+%28Daytime+sleep%5BTitle%2FAbstract%5D%29%29+OR+%28Siesta%5BTitle%2FAbstract%5D%29%29+OR+%28Daytime+sleepiness%5BTitle%2FAbstract%5D%29%29+OR+%28Daytime+somnolence%5BTitle%2FAbstract%5D%29%29+OR+%28Dozing%5BTitle%2FAbstract%5D%29%29+OR+%28Catnap%5BTitle%2FAbstract%5D%29%29+OR+%28Snooze%5BTitle%2FAbstract%5D%29&sort=relevance&size=200) |
| #2 | Search: "Mortality"[Mesh] Sort by: Most Recent | [426.757](https://pubmed.ncbi.nlm.nih.gov/?term=%28%22Diabetes+Mellitus%22%5BMesh%5D%29+OR+%28%28%28%28%28%28%28Glucose+Metabolism+Disorders%5BTitle%2FAbstract%5D%29+OR+%28Diabetes+Mellitus%5BTitle%2FAbstract%5D%29%29+OR+%28Wolfram+Syndrome%5BTitle%2FAbstract%5D%29%29+OR+%28Diabet%2A%5BTitle%2FAbstract%5D%29%29+OR+%28Donohue+Syndrome%5BTitle%2FAbstract%5D%29%29+OR+%28Prediabetic%5BTitle%2FAbstract%5D%29%29+OR+%28Glycemic+control%5BTitle%2FAbstract%5D%29%29&sort=date&size=200&ac=no) |
| #3 | Search: (((((((((Mortality[Title/Abstract]) OR (Death[Title/Abstract])) OR (suicide[Title/Abstract])) OR (Case Fatality[Title/Abstract])) OR (Crude Death[Title/Abstract])) OR (Mortality Decline[Title/Abstract])) OR (Mortality Determinant[Title/Abstract])) OR (Differential Mortality[Title/Abstract])) OR (Age-Specific Death Rate[Title/Abstract])) OR (Excess Mortality[Title/Abstract]) | [1,851,185](https://pubmed.ncbi.nlm.nih.gov/?term=%28%28%28%28%28%28%28%28%28Mortality%5BTitle%2FAbstract%5D%29+OR+%28Death%5BTitle%2FAbstract%5D%29%29+OR+%28suicide%5BTitle%2FAbstract%5D%29%29+OR+%28Case+Fatality%5BTitle%2FAbstract%5D%29%29+OR+%28Crude+Death%5BTitle%2FAbstract%5D%29%29+OR+%28Mortality+Decline%5BTitle%2FAbstract%5D%29%29+OR+%28Mortality+Determinant%5BTitle%2FAbstract%5D%29%29+OR+%28Differential+Mortality%5BTitle%2FAbstract%5D%29%29+OR+%28Age-Specific+Death+Rate%5BTitle%2FAbstract%5D%29%29+OR+%28Excess+Mortality%5BTitle%2FAbstract%5D%29&sort=relevance&size=200) |
| #4 | Search: ("Mortality"[Mesh]) OR ((((((((((Mortality[Title/Abstract]) OR (Death[Title/Abstract])) OR (suicide[Title/Abstract])) OR (Case Fatality[Title/Abstract])) OR (Crude Death[Title/Abstract])) OR (Mortality Decline[Title/Abstract])) OR (Mortality Determinant[Title/Abstract])) OR (Differential Mortality[Title/Abstract])) OR (Age-Specific Death Rate[Title/Abstract])) OR (Excess Mortality[Title/Abstract])) | [2,067,730](https://pubmed.ncbi.nlm.nih.gov/?term=%28%22Mortality%22%5BMesh%5D%29+OR+%28%28%28%28%28%28%28%28%28%28Mortality%5BTitle%2FAbstract%5D%29+OR+%28Death%5BTitle%2FAbstract%5D%29%29+OR+%28suicide%5BTitle%2FAbstract%5D%29%29+OR+%28Case+Fatality%5BTitle%2FAbstract%5D%29%29+OR+%28Crude+Death%5BTitle%2FAbstract%5D%29%29+OR+%28Mortality+Decline%5BTitle%2FAbstract%5D%29%29+OR+%28Mortality+Determinant%5BTitle%2FAbstract%5D%29%29+OR+%28Differential+Mortality%5BTitle%2FAbstract%5D%29%29+OR+%28Age-Specific+Death+Rate%5BTitle%2FAbstract%5D%29%29+OR+%28Excess+Mortality%5BTitle%2FAbstract%5D%29%29&sort=relevance&size=200) |
| #5 | Search: "Cardiovascular Diseases"[Mesh] Sort by: Most Recent | [2,782,935](https://pubmed.ncbi.nlm.nih.gov/?sort=date&term=%22Cardiovascular+Diseases%22%5BMesh%5D&size=200) |
| #6 | Search: (((((((((((Cardiovascular Diseases[Title/Abstract]) OR (Cardiac Event[Title/Abstract])) OR (Cardiomyopathy[Title/Abstract])) OR (heart failure[Title/Abstract])) OR (cerebrovascular disease[Title/Abstract])) OR (peripheral vascular disease[Title/Abstract])) OR (coronary heart disease[Title/Abstract])) OR (ischemic heart disease[Title/Abstract])) OR (myocardial infarction[Title/Abstract])) OR (stroke[Title/Abstract])) OR (heart attack[Title/Abstract])) OR (hypertension[Title/Abstract]) | [1,294,076](https://pubmed.ncbi.nlm.nih.gov/?term=%28%28%28%28%28%28%28%28%28%28%28Cardiovascular+Diseases%5BTitle%2FAbstract%5D%29+OR+%28Cardiac+Event%5BTitle%2FAbstract%5D%29%29+OR+%28Cardiomyopathy%5BTitle%2FAbstract%5D%29%29+OR+%28heart+failure%5BTitle%2FAbstract%5D%29%29+OR+%28cerebrovascular+disease%5BTitle%2FAbstract%5D%29%29+OR+%28peripheral+vascular+disease%5BTitle%2FAbstract%5D%29%29+OR+%28coronary+heart+disease%5BTitle%2FAbstract%5D%29%29+OR+%28ischemic+heart+disease%5BTitle%2FAbstract%5D%29%29+OR+%28myocardial+infarction%5BTitle%2FAbstract%5D%29%29+OR+%28stroke%5BTitle%2FAbstract%5D%29%29+OR+%28heart+attack%5BTitle%2FAbstract%5D%29%29+OR+%28hypertension%5BTitle%2FAbstract%5D%29&sort=relevance&size=200) |
| #7 | Search: ("Cardiovascular Diseases"[Mesh]) OR ((((((((((((Cardiovascular Diseases[Title/Abstract]) OR (Cardiac Event[Title/Abstract])) OR (Cardiomyopathy[Title/Abstract])) OR (heart failure[Title/Abstract])) OR (cerebrovascular disease[Title/Abstract])) OR (peripheral vascular disease[Title/Abstract])) OR (coronary heart disease[Title/Abstract])) OR (ischemic heart disease[Title/Abstract])) OR (myocardial infarction[Title/Abstract])) OR (stroke[Title/Abstract])) OR (heart attack[Title/Abstract])) OR (hypertension[Title/Abstract])) | [3,184,289](https://pubmed.ncbi.nlm.nih.gov/?term=%28%22Cardiovascular+Diseases%22%5BMesh%5D%29+OR+%28%28%28%28%28%28%28%28%28%28%28%28Cardiovascular+Diseases%5BTitle%2FAbstract%5D%29+OR+%28Cardiac+Event%5BTitle%2FAbstract%5D%29%29+OR+%28Cardiomyopathy%5BTitle%2FAbstract%5D%29%29+OR+%28heart+failure%5BTitle%2FAbstract%5D%29%29+OR+%28cerebrovascular+disease%5BTitle%2FAbstract%5D%29%29+OR+%28peripheral+vascular+disease%5BTitle%2FAbstract%5D%29%29+OR+%28coronary+heart+disease%5BTitle%2FAbstract%5D%29%29+OR+%28ischemic+heart+disease%5BTitle%2FAbstract%5D%29%29+OR+%28myocardial+infarction%5BTitle%2FAbstract%5D%29%29+OR+%28stroke%5BTitle%2FAbstract%5D%29%29+OR+%28heart+attack%5BTitle%2FAbstract%5D%29%29+OR+%28hypertension%5BTitle%2FAbstract%5D%29%29&sort=relevance&size=200) |
|  | Search: ((((((((((Napping[Title/Abstract]) OR (Nap[Title/Abstract])) OR (Daytime sleep[Title/Abstract])) OR (Siesta[Title/Abstract])) OR (Daytime sleepiness[Title/Abstract])) OR (Daytime somnolence[Title/Abstract])) OR (Dozing[Title/Abstract])) OR (Catnap[Title/Abstract])) OR (Snooze[Title/Abstract])) AND (("Mortality"[Mesh]) OR ((((((((((Mortality[Title/Abstract]) OR (Death[Title/Abstract])) OR (suicide[Title/Abstract])) OR (Case Fatality[Title/Abstract])) OR (Crude Death[Title/Abstract])) OR (Mortality Decline[Title/Abstract])) OR (Mortality Determinant[Title/Abstract])) OR (Differential Mortality[Title/Abstract])) OR (Age-Specific Death Rate[Title/Abstract])) OR (Excess Mortality[Title/Abstract])))) AND (("Cardiovascular Diseases"[Mesh]) OR ((((((((((((Cardiovascular Diseases[Title/Abstract]) OR (Cardiac Event[Title/Abstract])) OR (Cardiomyopathy[Title/Abstract])) OR (heart failure[Title/Abstract])) OR (cerebrovascular disease[Title/Abstract])) OR (peripheral vascular disease[Title/Abstract])) OR (coronary heart disease[Title/Abstract])) OR (ischemic heart disease[Title/Abstract])) OR (myocardial infarction[Title/Abstract])) OR (stroke[Title/Abstract])) OR (heart attack[Title/Abstract])) OR (hypertension[Title/Abstract]))) | [312](https://pubmed.ncbi.nlm.nih.gov/?term=%28%28%28%28%28%28%28%28%28%28Napping%5BTitle%2FAbstract%5D%29+OR+%28Nap%5BTitle%2FAbstract%5D%29%29+OR+%28Daytime+sleep%5BTitle%2FAbstract%5D%29%29+OR+%28Siesta%5BTitle%2FAbstract%5D%29%29+OR+%28Daytime+sleepiness%5BTitle%2FAbstract%5D%29%29+OR+%28Daytime+somnolence%5BTitle%2FAbstract%5D%29%29+OR+%28Dozing%5BTitle%2FAbstract%5D%29%29+OR+%28Catnap%5BTitle%2FAbstract%5D%29%29+OR+%28Snooze%5BTitle%2FAbstract%5D%29%29+AND+%28%28%22Mortality%22%5BMesh%5D%29+OR+%28%28%28%28%28%28%28%28%28%28Mortality%5BTitle%2FAbstract%5D%29+OR+%28Death%5BTitle%2FAbstract%5D%29%29+OR+%28suicide%5BTitle%2FAbstract%5D%29%29+OR+%28Case+Fatality%5BTitle%2FAbstract%5D%29%29+OR+%28Crude+Death%5BTitle%2FAbstract%5D%29%29+OR+%28Mortality+Decline%5BTitle%2FAbstract%5D%29%29+OR+%28Mortality+Determinant%5BTitle%2FAbstract%5D%29%29+OR+%28Differential+Mortality%5BTitle%2FAbstract%5D%29%29+OR+%28Age-Specific+Death+Rate%5BTitle%2FAbstract%5D%29%29+OR+%28Excess+Mortality%5BTitle%2FAbstract%5D%29%29%29%29+AND+%28%28%22Cardiovascular+Diseases%22%5BMesh%5D%29+OR+%28%28%28%28%28%28%28%28%28%28%28%28Cardiovascular+Diseases%5BTitle%2FAbstract%5D%29+OR+%28Cardiac+Event%5BTitle%2FAbstract%5D%29%29+OR+%28Cardiomyopathy%5BTitle%2FAbstract%5D%29%29+OR+%28heart+failure%5BTitle%2FAbstract%5D%29%29+OR+%28cerebrovascular+disease%5BTitle%2FAbstract%5D%29%29+OR+%28peripheral+vascular+disease%5BTitle%2FAbstract%5D%29%29+OR+%28coronary+heart+disease%5BTitle%2FAbstract%5D%29%29+OR+%28ischemic+heart+disease%5BTitle%2FAbstract%5D%29%29+OR+%28myocardial+infarction%5BTitle%2FAbstract%5D%29%29+OR+%28stroke%5BTitle%2FAbstract%5D%29%29+OR+%28heart+attack%5BTitle%2FAbstract%5D%29%29+OR+%28hypertension%5BTitle%2FAbstract%5D%29%29%29&sort=relevance&size=200) |

Table 2 Embase

| No. | Content | Result |
| --- | --- | --- |
| #1 | 'napping'/exp OR napping OR nap:ab,ti OR 'daytime sleep':ab,ti OR siesta:ab,ti OR 'daytime sleepiness':ab,ti OR 'daytime somnolence':ab,ti OR dozing:ab,ti OR catnap:ab,ti OR snooze:ab,ti | 29,555 |
| #2 | 'mortality'/exp | 1,459,633 |
| #3 | 'mortality'/exp OR mortality OR death:ab,ti OR 'daytime sleep':ab,ti OR 'case fatality':ab,ti OR 'crude death':ab,ti OR 'mortality decline':ab,ti OR 'mortality determinant':ab,ti OR 'differential mortality':ab,ti OR 'age-specific death rate':ab,ti OR 'excess mortality':ab,ti | 2,995,778 |
| #4 | #2 OR #3 | 2,995,778 |
| #5 | 'cardiovascular disease'/exp | 5,650,442 |
| #6 | 'cardiovascular diseases'/exp OR 'cardiovascular diseases' OR (('cardiovascular'/exp OR cardiovascular) AND ('diseases'/exp OR diseases)) OR 'cardiac event':ab,ti OR cardiomyopathy:ab,ti OR 'heart failure':ab,ti OR 'cerebrovascular disease':ab,ti OR 'peripheral vascular disease':ab,ti OR 'coronary heart disease':ab,ti OR 'ischemic heart disease':ab,ti OR 'myocardial infarction':ab,ti OR stroke:ab,ti OR 'heart attack':ab,ti OR hypertension:ab,ti | 6,164,629 |
| #7 | #5 OR #6 | 6,164,629 |
| #8 | #2 AND #4 AND #7 | 893 |

Table 3 Cochrane Library

| No. | Content | Result |
| --- | --- | --- |
| #1 | (Napping):ti,ab,kw OR (Nap):ti,ab,kw OR (Daytime sleep):ti,ab,kw OR (Siesta):ti,ab,kw OR (Daytime sleepiness):ti,ab,kw | 5,876 |
| #2 | (Daytime somnolence):ti,ab,kw OR (Dozing):ti,ab,kw OR (Catnap):ti,ab,kw OR (Snooze):ti,ab,kw | 1,580 |
| #3 | #1 OR #2 | 5,976 |
| #4 | MeSH descriptor: [Mortality] explode all trees | 18,869 |
| #5 | (Mortality):ti,ab,kw OR (Death):ti,ab,kw OR (suicide):ti,ab,kw OR (Case Fatality):ti,ab,kw OR (Crude Death):ti,ab,kw | 177,793 |
| #6 | (Mortality Decline):ti,ab,kw OR (Mortality Determinant):ti,ab,kw OR (Differential Mortality):ti,ab,kw OR (Age-Specific Death Rate):ti,ab,kw OR (Excess Mortality):ti,ab,kw | 180,995 |
| #7 | #4 OR #5 OR #6 | 25,564 |
| #8 | MeSH descriptor: [Cardiovascular Diseases] explode all trees | 156,115 |
| #9 | (Cardiovascular Diseases):ti,ab,kw OR (Cardiac Event):ti,ab,kw OR (Cardiomyopathy):ti,ab,kw OR (heart failure):ti,ab,kw OR (cerebrovascular disease):ti,ab,kw | 119,045 |
| #10 | (peripheral vascular disease):ti,ab,kw OR (coronary heart disease):ti,ab,kw OR (ischemic heart disease):ti,ab,kw OR (myocardial infarction):ti,ab,kw OR (stroke):ti,ab,kw | 123,516 |
| #11 | (heart attack):ti,ab,kw OR (hypertension):ti,ab,kw | 83,048 |
| #12 | #8 OR #9 OR #10 OR #11 | 307,441 |
| #13 | #3 AND #7 AND #12 | 147 |

Table 4 Web of science

| No. | Content | Result |
| --- | --- | --- |
| #1 | Napping (Topic) or Nap (Topic) or Daytime sleep (Topic) or Daytime sleepiness (Topic) or Siesta (Topic) or Daytime somnolence (Topic) or Dozing (Topic) or carnap (Topic) or sneeze (Topic) | [43,595](https://www.webofscience.com/wos/woscc/summary/84f45cec-c44d-45b9-b4a2-b6aca68badba-dedf4267/relevance/1) |
| #2 | Mortality (Topic) or Death (Topic) or suicide (Topic) or Case Fatality (Topic) or Crude Death (Topic) or Mortality Decline (Topic) or Mortality Determinant (Topic) or Differential Mortality (Topic) or Age-Specific Death Rate (Topic) or Excess Mortality (Topic) | 2,435,277 |
| #3 | Cardiovascular Diseases (Topic) or Cardiac Event (Topic) or Cardiomyopathy (Topic) or heart failure (Topic) or cerebrovascular disease (Topic) or peripheral vascular disease (Topic) or coronary heart disease (Topic) or ischemic heart disease (Topic) or myocardial infarction (Topic) or stroke (Topic) or heart attack (Topic) or hypertension (Topic) | 1,982,373 |
| #4 | #1 AND #2 AND #3 | 1,050 |
