# Supplementary material for: Association between self-reported napping and risk of cardiovascular disease and all-cause mortality: A meta-analysis of cohort studies
Source: PLoS One. 2024 Oct 16;19(10):e0311266. doi: 10.1371/journal.pone.0311266 (PMC11482734; doi:10.1371/journal.pone.0311266)
Supplement: S5 Table — (DOCX) [file pone.0311266.s005.docx]

**Table S5. The quality assessment of cohort and case-control studies.**

| Study | Year | Selection | Comparability | Outcome | Total |
| --- | --- | --- | --- | --- | --- |
| Cohort studies (n=21) | | | | | |
| Hays, J. C.et al | 1996 | ** | ** | ** | 6 |
| Bursztyn, M | 1999 | ** | ** | ** | 6 |
| Bursztyn, M. | 2002 | ** | ** | ** | 6 |
| Naska, A. et al | 2007 | *** | ** | ** | 7 |
| Stone, K. L. et al | 2009 | *** | ** | *** | 8 |
| Tanabe, N. et al | 2010 | *** | ** | *** | 8 |
| Stang, A. et al | 2012 | *** | ** | *** | 8 |
| Leng, Y.et al | 2014 | *** | ** | *** | 8 |
| Wannamethee, S. G. et al | 2016 | *** | ** | *** | 8 |
| Zhou, J. M. et al | 2016 | *** | ****** | ****** | **7** |
| Wang, C.S et al | 2017 | ** | ** | ** | 6 |
| Xiao, Q. et al | 2017 | **** | ** | *** | 9 |
| Häusler, N. et al | 2019 | *** | ** | *** | 8 |
| Yan, B. et al | 2019 | *** | ** | *** | 8 |
| Wang, L. et al | 2022 | *** | ** | *** | 8 |
| Wang, Z. Y. et al | 2022 | *** | ** | *** | 8 |
| Chen, A. et al | 2023 | *** | ** | *** | 8 |
| Diao, T. Y. et al | 2023 | *** | ** | *** | 8 |
| Ke, W. et al | 2023 | *** | ** | *** | 8 |
| Wang, L.et al | 2023 | *** | ** | ** | 7 |
| Zhang, Y. T. et al | 2024 | *** | ** | *** | 8 |

The NOS scale was used to evaluate the quality of the cohort and case-control studies.
