# Supplementary material for: Association between self-reported napping and risk of cardiovascular disease and all-cause mortality: A meta-analysis of cohort studies
Source: PLoS One. 2024 Oct 16;19(10):e0311266. doi: 10.1371/journal.pone.0311266 (PMC11482734; doi:10.1371/journal.pone.0311266)
Supplement: S6 Table — (DOCX) [file pone.0311266.s006.docx]

**Sensitivity analysis**

1. Napping and non-napping in relation to the risk of all-cause mortality

Deleting any literature from this study will not affect the results, indicating that the calculation results of the random effects are stable and reliable. As shown in the figure below:

2.Napping less than 1 hour and non-napping in relation to the risk of all-cause mortality

Deleting any literature from this study will not affect the results, indicating that the calculation results of the random effects are stable and reliable. As shown in the figure below:

3. Napping 1 hour or longer and non-napping in relation to the risk of all-cause mortality.

Deleting any literature from this study will not affect the results, indicating that the calculation results of the random effects are stable and reliable. As shown in the figure below:

4. Daytime napping and non-napping in relation to CVD risk

Deleting any literature from this study will not affect the results, indicating that the calculation results of the random effects are stable and reliable. As shown in the figure below:

5. Napping less than 1 hour and non-napping in relation to CVD risk

Deleting any literature from this study will not affect the results, indicating that the calculation results of the random effects are stable and reliable. As shown in the figure below:

6. Napping 1 hour or longer and non-napping in relation to CVD risk.

Deleting any literature from this study will not affect the results, indicating that the calculation results of the random effects are stable and reliable. As shown in the figure below:
